# Investigation of the Albumin/Globulin Ratio as a Biomarker in Restless Legs Syndrome

**DOI:** 10.7759/cureus.51671

**Published:** 2024-01-04

**Authors:** Esen Çiçekli, Elif Sarıca Darol

**Affiliations:** 1 Neurology, Akyazi State Hospital, Sakarya, TUR; 2 Neurology, Sakarya University Training and Research Hospital, Sakarya, TUR

**Keywords:** sleep disorders, agr, globulin, albumin, restless legs syndrome

## Abstract

Background

Measurement of serum/plasma levels of inflammatory factors in restless legs syndrome (RLS) has been the subject of a few studies, as various inflammatory diseases may demonstrate an association with RLS. The albumin/globulin ratio (AGR) is a parameter that considers two proteins that are indicators of inflammation and has been shown to increase in some inflammatory diseases. No study has evaluated the relationship between RLS and AGR yet. In our study, we examined the usability of AGR as a diagnostic biomarker in RLS patients.

Methodology

A total of 88 patients and 89 control individuals were included in the study retrospectively. The two groups were compared in terms of AGR levels. RLS patients were divided into four groups according to the International Restless Legs Syndrome Study Group Rating Scale score and the relationship between disease severity and AGR values ​​was examined.

Results

Albumin levels of the study group were higher than the control group. There was no statistically significant difference between the groups regarding globulin and AGR levels.

Conclusions

Serum albumin levels could be useful in RLS compared to globulin or AGR values. This needs to be supported by new studies with larger patient series, multicenter design, and including multiple parameters such as patients’ muscle mass, nutritional habits, and exercise status.

## Introduction

Restless legs syndrome (RLS) is a chronic sensorimotor disorder diagnosed mostly based on clinical symptoms [1]. The frequency of the RLS has been reported to be between 0.1% and 15% in different studies. It is more common in women than men [2,3]. It can be defined by patients as irritating symptoms such as pins and needles, stinging, burning, stretching, and indescribable restlessness. The symptoms are often unbearable, and patients seek remedies to alleviate their symptoms, such as moving or massaging their extremities.

The etiology of RLS is not known, but the currently accepted pathways include genetic predisposition, iron dysregulation in the central nervous system, and dysfunction in the dopaminergic and nociceptive systems [4,5]. It is thought that RLS is a complex condition that occurs due to predisposing genetic factors, with the contribution of some environmental factors and comorbidities. RLS can occur alone or in combination with iron deficiency, kidney disease, cardiovascular diseases, diabetes, neurological diseases, rheumatological diseases, and respiratory disorders [6].

RLS is often poorly recognized or misdiagnosed in clinical practice. Common pharmacological treatments are iron preparations, dopaminergic agents, alpha 2 delta ligands, and opioids. Although dopaminergic therapy is initially quite effective, its long-term use can cause severe worsening of symptoms, known as augmentation. Other treatments include iron preparations, alpha 2 delta ligands, and opioids [6,7]. The fact that the etiology of RLS is not fully known and that it has a wide differential diagnosis has led researchers to search for various biomarkers. Measurement of serum/plasma levels of inflammatory factors has been the subject of few studies, as various inflammatory diseases may demonstrate an association with RLS. A limited number of studies on the relationship between inflammatory molecules such as C-reactive protein, neutrophils, lymphocytes, albumin, and RLS have yielded findings that inflammatory changes may be effective on the etiology of RLS [8,9].

Albumin gives information not only about nutrition but also about the inflammatory state in the body. Globulin consists of many proteins associated with inflammation, and its levels increase in inflammatory events. The albumin/globulin ratio (AGR) takes both albumin and globulin into account to give a more accurate indication of the body’s nutritional and inflammatory status [10]. Low AGR has been associated with poor prognosis in diseases associated with inflammation such as some cancers and dialysis patients [11,12]. In our study, we aimed to evaluate the AGR ratio, which can provide information about both muscle mass and inflammation status, in RLS patients and to investigate the usability of AGR as a biomarker in the diagnosis of RLS.

## Materials and methods

This is a retrospective clinical study. Ethics committee approval for the study was received from the Sakarya University Faculty of Medicine Ethics Committee (approval number: E-71522473-050.01.04-285275-281). The patient and control groups were informed about the study and their consent was obtained.

The files of patients diagnosed with RLS who applied to the neurology outpatient clinic between April 2022 and April 2023 were retrospectively examined by the physicians. A total of 88 patients who met the inclusion criteria were included in the study. As a control group, 89 individuals who were not diagnosed with RLS and met the criteria were included in the study. The files of the patients to be included in the control group were also examined retrospectively.

Demographic information, anamnesis, examination findings, serum magnesium, ferritin, albumin, globulin, and AGR values of the patient and control groups were recorded from the hospital automation system. The laboratory results checked at the participants’ first application were included in the study. The mentioned parameters were compared in the patient and control groups and examined to determine if there was a significant difference.

Using the International Restless Legs Syndrome Study Group rating scale (IRLS) for RLS, patients were divided into four groups according to the severity of symptoms [13]. Those with a score of 1-10 were grouped as mild, 11-20 were grouped as moderate, 21-30 were grouped as severe, and 31-40 were grouped as very severe. Differences between these groups in terms of the mentioned parameters were determined. The inclusion criteria and exclusion criteria are presented in Table 1.

**Table 1:** Inclusion and exclusion criteria. RLS: restless legs syndrome

Inclusion criteria	Exclusion criteria
Having been diagnosed with RLS according to the criteria of the International Restless Legs Working Group [13]	Individuals who have received albumin replacement in the last three months for any reason
Between the ages of 18 and 80 years	Having a disease that affects the albumin/globulin level (kidney diseases, liver diseases, severe malnutrition, etc.)

Statistical analysis

SPSS Statistics version 27.0 (IBM Corp., Armonk, NY, USA) was used for data analysis. In the evaluation of the research data, mean, standard deviation, frequency, minimum, and maximum values were used as descriptive statistical methods. The skewness and kurtosis (±1.5) distribution test was used to examine the normal distribution. It was determined that the data showed normal distribution. For statistical calculations, the independent t-test was used for the significance of the difference between the means in independent groups. Analysis of variance was used to compare more than two groups, and Pearson correlation analysis was used to evaluate the significance of the relationship between parameters.

## Results

Our study included 88 patients, 69 women and 19 men, and 89 control individuals, 68 women and 21 men. The mean age of the study group was 58.65 ± 12.18 years, and the mean age of the control group was 56.26 ± 17.82 years. There was no statistical difference between the groups according to the mean age.

There was a statistically significant difference between the study group and the control group regarding magnesium, ferritin, and albumin levels. The study group had higher magnesium and albumin levels and lower ferritin levels. There was no statistically significant difference between the groups in terms of globulin and AGR levels (Table 2).

**Table 2:** Age, magnesium, ferritin, albumin, globulin, and AGR values of the study and control groups. AGR: albumin/globulin ratio; Min: minimum; Max: maximum; SD: standard deviation

	Study group			Control group			
Parameter	Min	Max	Mean ± SD	Min	Max	Mean ± SD	P-value
Age	30	80	58.65 ± 12.18	18	80	56.26 ± 17.82	0.301
Magnesium (mg/dL)	1.30	2.2	1.89 ± 0.16	0.8	2.4	1.71 ± 0.31	0.001*
Ferritin (ng/mL)	1.15	269	29.26 ± 34.04	3.6	411.2	47.23 ± 58.05	0.013*
Albumin (g/dL)	36.0	48.5	42.87 ± 2.61	28.8	52.5	41.29 ± 3.61	0.001*
Globulin (mg/dL)	12.9	48.3	28.60 ± 5.18	18.5	46	28.66 ± 4.21	0.933
AGR	0.88	3.36	1.55 ± 0.33	0.63	2.26	1.47 ± 0.26	0.089

Individuals in the study and control groups were divided into two groups as ferritin levels below and above 30 ng/mL. Ferritin levels of 56 individuals in the study group and 44 individuals in the control group were below 30 ng/mL (Table 3).

**Table 3:** Distribution of individuals with a ferritin value above and below 30 ng/mL by groups.

Serum ferritin level	Study group (n)	Control group (n)
Ferritin <30 ng/mL	56	44
Ferritin >30 ng/mL	32	45
Total (N)	88	89

The distribution of the patients grouped according to IRLS is shown in Table 4. A homogeneity test was performed between groups. The groups were homogeneously distributed (p > 0.05).

**Table 4:** Distribution of participants according to IRLS. IRLS: International Restless Legs Syndrome Study Group Rating Scale

Groups	N
Mild	0
Moderate	19
Severe	44
Very severe	25
Total	88

The significance of the difference between IRLS groups was tested by analysis of variance. According to IRLS, there was no significant difference between the groups in terms of magnesium, ferritin, albumin, globulin, and AGR (p > 0.05) (Table 5). Post-hoc test was not performed as there was no difference between the groups.

**Table 5:** Evaluation of the difference between disease severity groups in terms of magnesium, ferritin, albumin, globulin, and AGR according to the IRLS score. AGR: albumin/globulin ratio; IRLS: International Restless Legs Syndrome Study Group Rating Scale

Parameter	F	P-value
Magnesium (mg/dL)	1.799	0.172
Ferritin (ng/mL)	0.110	0.896
Albumin (g/dL)	0.155	0.857
Globulin (mg/dL)	0.234	0.792
AGR	0.248	0.781

## Discussion

In our study, a significant difference was found between the patient and control groups in terms of magnesium, ferritin, and albumin values.

The patient group had higher magnesium levels than the control group. Although magnesium deficiency may be an underlying cause of RLS according to some studies, its usability in the treatment of RLS remains controversial [14,15]. In our country, magnesium preparations were used quite often by patients without a doctor’s prescription to relieve various symptoms. Especially patients in the mild group applied similar medications themselves before consulting a doctor. Although its definitive usefulness has not been shown in various studies, magnesium is prescribed to patients by family physicians to relieve their symptoms, especially cramps and spasms [16]. Therefore, some of the patients who consult a neurologist have previously used magnesium. We did not exclude patients receiving magnesium, and this situation could lead to different results.

Ferritin levels of the RLS patients in our study were significantly lower than the control group. The number of participants with ferritin levels below 30 was also higher in the patient group. In most studies, RLS is associated with low ferritin levels, and it is emphasized that iron preparations are useful in treatment [17]. Various pathological studies or brain imaging studies have found iron deficiency in the brain and cerebrospinal fluid of patients with RLS. There is no strong evidence regarding the cause of this deficiency [18].

Although there was no correlation between disease severity and albumin level in RLS patients, albumin levels in the patient group were statistically significantly higher than in the control group. Based on this result, albumin levels may be associated with the presence of RLS disease. While albumin can be used as a biomarker that increases during inflammation, according to some studies, it may also be related to body muscle mass, dietary amino acid intake, and exercise status [10,19]. Additionally, another study found a relationship between low physical activity and RLS [20]. In this context, our study will be a guide for new studies to examine the relationship between RLS and physical activity, muscle mass, and nutritional status.

When RLS patients and the control group were compared, there was no difference between the two groups in terms of globulin levels or AGR values. In addition, these two parameters did not correlate with disease severity.

Although few studies have addressed the relationship between albumin and RLS, there are no studies in the literature investigating the relationship between globulin, AGR, and RLS. Hence, our study is the first on this subject and we believe that it will guide future research.

This study has a few limitations. It was conducted in a single center with a small number of patients, and parameters that may affect albumin levels, such as the nutrition and exercise status of the patients, were not included in the study.

## Conclusions

Serum albumin levels can be useful in RLS compared to globulin or AGR values. This needs to be supported by new studies with larger patient series with a multicenter design, as well as including multiple parameters such as patients’ muscle mass, nutritional habits, and exercise status.
